# *G6PD* distribution in sub-Saharan Africa and potential risks of using chloroquine/hydroxychloroquine based treatments for COVID-19

**DOI:** 10.1038/s41397-021-00242-8

**Published:** 2021-07-23

**Authors:** Jorge E. B. da Rocha, Houcemeddine Othman, Caroline T. Tiemessen, Gerrit Botha, Michèle Ramsay, Collen Masimirembwa, Clement Adebamowo, Ananyo Choudhury, Jean-Tristan Brandenburg, Mogomotsi Matshaba, Gustave Simo, Francisco-Javier Gamo, Scott Hazelhurst, as members of the H3Africa Consortium, Jorge E. B. da Rocha

**Affiliations:** 1 Sydney Brenner Institute for Molecular Bioscience, Faculty of Health Sciences, University of the Witwatersrand, Johannesburg, South Africa; 2 Division of Human Genetics, National Health Laboratory Service and School of Pathology, Faculty of Health Sciences, University of the Witwatersrand, Johannesburg, South Africa; 3 Centre for HIV and STIs, National Institute for Communicable Diseases, National Health Laboratory Services and Faculty of Health Sciences, University of the Witwatersrand, Johannesburg, South Africa; 4 Computational Biology Division and H3ABioNet, Department of Integrative Biomedical Sciences, University of Cape Town, Cape Town, South Africa; 5 Institute for Human Virology Abuja, Abuja, Nigeria; 6 Institute of Human Virology and Greenebaum Comprehensive Cancer Center, University of Maryland School of Medicine, Baltimore, MD USA; 7 Botswana-Baylor Children’s Clinical Center of Excellence, Gaborone, Botswana; 8 Baylor College of Medicine, Houston, TX USA; 9 Molecular Parasitology and Entomology Unit, Department of Biochemistry, Faculty of Science, University of Dschang, Dschang, Cameroon; 10 Global Health, GlaxoSmithKline R&D, Tres Cantos, Madrid, Spain; 11 School of Electrical & Information Engineering, University of the Witwatersrand, Johannesburg, South Africa

**Keywords:** Predictive medicine, Genetics research

## Abstract

Chloroquine/hydroxychloroquine have been proposed as potential treatments for COVID-19. These drugs have warning labels for use in individuals with glucose-6-phosphate dehydrogenase (G6PD) deficiency. Analysis of whole genome sequence data of 458 individuals from sub-Saharan Africa showed significant *G6PD* variation across the continent. We identified nine variants, of which four are potentially deleterious to G6PD function, and one (rs1050828) that is known to cause G6PD deficiency. We supplemented data for the rs1050828 variant with genotype array data from over 11,000 Africans. Although this variant is common in Africans overall, large allele frequency differences exist between sub-populations. African sub-populations in the same country can show significant differences in allele frequency (e.g. 16.0% in Tsonga vs 0.8% in Xhosa, both in South Africa, *p* = 2.4 × 10^*−*3^). The high prevalence of variants in the *G6PD* gene found in this analysis suggests that it may be a significant interaction factor in clinical trials of chloroquine and hydroxychloroquine for treatment of COVID-19 in Africans.

## Introduction

Chloroquine and hydroxychloroquine (CQ/HCQ) have been undergoing clinical trials as treatments for Coronavirus disease (COVID-19) which is caused by the severe acute respiratory syndrome coronavirus 2 (SARS-CoV-2) [1]. A clear mechanism of action of CQ/HCQ for SARS-CoV-2 treatment is yet to be determined. However, there are diverse hypothetical mechanisms which may result in prevention of viral entry into the cell, restriction of access to cell replication machinery, or by modulating the immunological response of the host (e.g cytokine storm) [2–4]. CQ/HCQ and other aminoquinolines have pharmacogenomic associations with the glucose-6-phosphate dehydrogenase (*G6PD*) gene [5]. Aminoquinolines are suspected to exert their antimalarial effect by increasing oxidative stress via production of haem-based reactive oxygen species [6]. The G6PD enzyme is responsible for the production of nicotinamide adenine dinucleotide phosphate (NADPH) which is required in the glutathione mediated detoxification of reactive oxygen species [7]. In the case of inactive/deficient G6PD, the NADPH supply may not be sufficient to neutralise the reactive oxygen species induced by CQ/HCQ and other drugs with similar mechanisms of action.

G6PD deficiency is common globally, particularly in African populations (14% of males) [8]. Individuals with the deficiency are at risk for haemolytic anaemia which can be triggered by infections, certain foods, or medications. G6PD deficiency is an X-linked disorder. It mostly occurs in males who are hemizygous for deleterious variants of the *G6PD* gene and in females with homozygous deleterious variants. Symptoms have also been observed in females with heterozygous combinations due to X-inactivation effects [9].

Three common haplotype arrangements have been defined for the gene, the B (wild type), A, and A– (deficiency) all of which are defined by a combination of variants from the rs1050829 and rs1050828 loci [5]. The *G6PD* A ‘haplotype’ is denoted by the rs1050829 C variant (NM_001042351.2:c.376 A > G). The rs1050829 C variant is not strongly linked to decreased G6PD activity and occurs in 10–40% of sub-Saharan Africans [10–12].

The A– haplotype (which is associated with G6PD enzyme deficiency) is formed by a combination of two variants, one of which is rs1050828 T (NM _001042351.2:c.202 G > A) (a deleterious variant), while the other is the rs1050829 C. This combination occurs in 10% of sub-Saharan Africans [5]. The A– *G6PD* haplotype is a World Health Organisation (WHO) Class III variant corresponding to a decrease from 10 to 60% of the normal enzyme activity [5, 13, 14]. Strong linkage disequilibrium exists between the rs1050828 T and rs1050829 C variants [15]. As the rs1050829 C allele is more common, rs1050828 T likely emerged after rs1050829 C, and then increased in frequency due to positive selection in Africans [16]. As rs1050829 C has a moderate effect on G6PD deficiency [11], it is reasonable to report G6PD deficiency based on rs1050828 genotype combinations alone.

The FDA has issued warnings on the use of CQ and HCQ in G6PD deficient individuals due to high risk of haemolytic anaemia, although these are not contraindicated [17, 18]. Acute haemolytic effects following CQ/HQC treatment for COVID-19 have been reported in at least three male cases [19–21], two of which are of African ancestry. Genetic testing confirmed the A– variant in the case presented by Kuipers et al. Whereas CQ is not known to induce severe haemolytic effects when used as an antimalarial in G6PD deficient individuals, in contrast to primaquine [22] or chloroproguanil [23], the risk of its therapeutic use in G6PD deficient, COVID-19 patients has been observed in case reports, but requires further study.

In this paper, we evaluate the prevalence of variants in G6PD gene in individuals of African ancestry. We suggest that variations in the *G6PD* gene could significantly affect risk of adverse effects of CQ/HCQ, and recommend that this should be evaluated in clinical trials of CQ/HCQ treatment for COVID-19. We also report the prevalence of a key *G6PD* variant, rs1050828, in 11,030 Africans from four countries in west, east and southern Africa, and show that not only is the variant allele common in Africa overall, but that there are very large differences between different groups, even between those who reside in close proximity.

## Methods

**Fig. 1:**
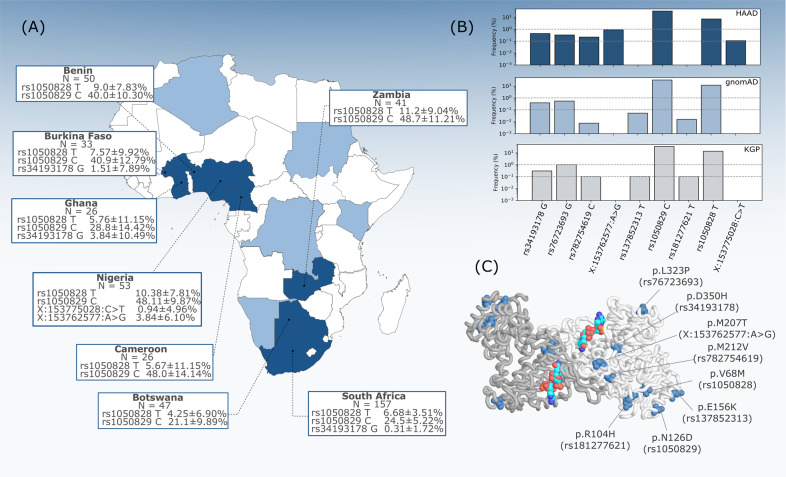
*G6PD* missense variant distribution across African populations. **A** G6PD allele frequencies in populations from high coverage African ADME Dataset (HAAD) countries. Confidence intervals for allele frequencies based on the equal or given proportions test the 95% significance level. Dark shading indicates country populations assessed, light shading indicates countries containing HAAD individuals but were not evaluated as individual countries due to fewer than 25 representative individuals. **B** Allele frequencies of missense variants in HAAD, and African superpopulation groups from gnomAD and the KGP. **C** Structural representation of the G6PD homodimer with missense residues highlighted in blue colour on both chains with bound NADP (NADP shown in red–turquoise–blue).

The dataset used was assembled as a collaborative project of the Human Heredity and Health in Africa (H3A) Consortium. The high coverage African ADME Dataset (HAAD) was sourced from H3A, other African collaborations and the Simons Foundation’s Genome Diversity Project [24]. HAAD consists of high-coverage sequences from 458 sub-Saharan African individuals from 15 countries, with 8 of these countries contributing data from more than 25 individuals (Nigeria, Ghana, Burkina Faso, Cameroon, Benin, Botswana, Zambia, and South Africa) (Fig. 1A). HAAD BAMS were aligned to GRCh37 with bwa-mem v0.7.10- v0.7.17 [25]. Variants were called with Haplotype-Caller in gVCF mode using GATK v.4.0.8.1. HAAD gVCFs (along with gVCFs produced with African 1000 Genomes Project data (KGA) [26]) were combined with GATK’s CombineGVCF (v.4.0.8.1), and jointly called with GenotypeGVCFs (v4.1.3.0) and followed GATK’s best practice guidelines. VQSR was used to select high quality sites with PASS ratings. All related workflows for data preparation can be found at https://github.com/h3abionet/recalling. The *G6PD* canonical gene region (chrX:153759606-153775469) was extracted with bcftools v1.9, and variants were annotated (e.g. as missense, intronic etc.) with variant effect predictor v92.0 [27] and SNPeff v4.3t [28]. Coding variants were selected for analysis if they meet a *QUAL* > 50 quality score. Functional annotation for these variants was performed using dbNSFP [29] to retrieve scores for five predictive toolsets (LRT, MutationAssessor, PROVEAN, VEST3 and CADD), which were then used for prediction based on a pharmacogene optimised model [30]. These form part of the “g_miner” workflow which is available at: https://github.com/hothman/PGx-Tools/tree/master/workflows/g_miner. PLINK [31] was used to call allele frequencies in every country in the HAAD dataset. Statistical analyses were conducted using R v3.63 [32]. The test for equal or given proportions was used to calculate allele frequency confidence intervals (CI) at the 95% significance level. Fisher’s exact test was used to assess significant differences in allele frequency between two populations, at the 5% significance threshold.

The impact of the variant on the protein structure was assessed by DynaMut [33] using the crystal structure of Canton G6PD modified to the wild type form [34]. Stability-related metrics calculated by Dynamut include the change in Gibbs free energy (∆∆*G*).

The A– haplotype in this study is defined by the presence of the rs1050828 T allele (alleles assessed in forward orientation, this corresponds to the c.202 G > A nomenclature for cDNA NM_001042351.2). The presence of rs1050829 C (corresponding to c.376 A > G, NM_001042351.2) is assumed due to strong linkage disequilibrium with rs1050828 T [15]. WHO classifications have been applied to *G6PD* variants according to the scale proposed by Yoshida et al. to reflect different levels of enzyme deficiency [13, 14]: I ≤ 10% and chronic anaemia; II ≤ 10% with risk of acute haemolytic anaemia; III = 10 < 60% and risk of acute haemolytic anaemia, IV = 60 < 100%.

The rs1050828 genotypes were also previously generated by the AWI-Gen Project [35] on 11,062 sub-Saharan Africans from 6 sites in Ghana, Burkina Faso, Kenya and South Africa using the H3A Custom Genotyping Array (https://www.h3abionet.org/h3africa-chip). PLINK [31] was used to remove any individuals with more than 1% missingness or genotypes which conflicted with declared sex. This left 11,030 individuals in all. The cluster plots of the genotype calls (male, female, all) are consistent with a well-genotyped single nucleotide polymorphism on the X chromosome. Minor allele frequency (MAF) was computed for the overall AWI-Gen data set as well as various sub-groups, as determined by self-identified ethnicity. The data was analysed using a custom Python script using the pandas-plink library (https://github.com/limix/pandas-plink).

## Results

### Variation from high coverage data

Nine coding (missense) variants were identified in the WGS, of which seven have been previously described, and have rsIDs allocated on dbSNP (nomenclature referred to by rsID throughout, as defined in Table 1). No loss of function type variants were detected. Figure 1B shows the distribution of the *G6PD* missense variants across African populations, along with comparisons to their overall frequency in the African data from the Genome Aggregation Database (gnomAD) [36] and the 1000 Genomes Project (KGP) [26]. Of these nine variants, seven have at least one prediction score from the pharmacogene model indicating deleterious impact. The variants rs1050829 C (chrX:g.153763492 T > C), and chrX:g.153775028 C > T had no predictive score reaching model cut-off criteria.

**Table 1:** *G6PD* missense variants detected within the HAAD and KGA population datasets and their relative stability effect (∆∆*G*) for G6PD protein.

Variant ID	Nucleotide	Amino Acid	∆∆*G* (kcal/mol)	Class
rs34193178 G	c.1048 G > C	D350H	0.374	N/A
rs76723693 G	c.968 T > C	L323P	−0.872	III
rs782754619 C	c.634 A > G	M212V	−1.147	N/A
rs181277621 T	c.311 G > A	R104H	−1.401	N/A
chrX:g.153762577 A > G	c.620 T > C	M207T	−1.145	N/A
chrX:g.153775028 C > T	c.58 G > A	N/A	N/A	N/A
rs137852313 T	c.466 G > A	E156K	0.539	III
rs1050829 C	c.376 A > G	N126D	0.887	IV
rs1050828 T	c.202 G > A	V68M	−1.347	III

Class refers to WHO classification.

Nucleotide positions based on cDNA NM_001042351.2 cDNA (chrX:g.153775028 C > T G58A is indicated by NM_000402.4). Amino acid positions based on NP_001035810.1.

*N/A* not available.

G6PD deficiency (A–), as defined by the rs1050828 T allele, is widely distributed across the African continent (Fig. 1A). There are notable differences in frequency across different groups, and we note that frequency does not necessarily correlate with the geographic location of the country. The highest frequency observed in HAAD populations was in Zambians (11.2 ± 9.04%, CI (95%) [2.16, 20.24]), whereas the lowest was in Batswana (4.25%, CI (95%) [0, 11.15]) their geographic neighbours. At the low sample number, this difference is not significant (*p* = 0.09115), and the confidence interval is large; however, we note that allele frequency is not uniform across these and other HAAD African groups.

Two other missense variants in HAAD, rs76723693 G (chrX:g.153761240 A > G) and rs34193178 G (chrX:g.153761160 C > G), have robust predictions as being functionally deleterious (all model tools in consensus). rs76723693 G is defined as part of the A– haplotype and although very rare in Africans, it has been characterised in other global populations [5]. The rs34193178 G variant is rare overall, but is found at a 1.5% (CI (95%) [0, 9.39]) in HAAD Burkina Faso and 3.84% (CI (95%) [0,14.33]) in HAAD Ghana populations. It is also present in HAAD South Africans at 0.3 ± 1.72% (CI (95%) [0, 2.02]).

The two variants chrX:g.153762577 A > G and chrX:g.153775028 C > T were not found in dbSNP151, and have not been reported in the gnomAD and the KGP databases (Fig. 1B). The chrX:g.153762577 A > G variant is only found in the HAAD Nigerian population, at 3.8% (CI (95%) [0, 9.9]), but not seen in the KGA Nigerian Esan or Yoruba populations. The chrX:g.153775028 C > T is a singleton variant, and is only present in pre-protein structures and is thus not displayed in Fig. 1C.

Four of the protein variants (p.V68M, p.E156K, p.R104H; corresponding respectively to gene substitutions rs1050828 T, rs137852313 T, rs1050829 C, rs181277621 T) are located in the co-enzyme domain; p.L323P (rs782754619 C) and p.M207T (chrX:g.153762577 A > G) belong to the *α* + *β* domain of G6PD buried in the protein core; and p.L323P (rs76723693 G) and p.D350H (rs34193178 G) are exposed to the solvent. All the corresponding amino acid residues (with the exception of p.E156K of the rs137852313 T variant) are either densely packed against other residues within the structure of G6PD, or they establish polar contacts that appear to stabilise local conformations of nearby segments. Structural predictions based on the calculation of ∆∆*G* (kcal/mol) (Table 1) show a destabilising effect for the amino acid substitution corresponding to variants: rs76723693 G, rs782754619 C, chrX:g.153762577 A > G, rs181277621 T, and rs1050828 T. The rs34193178 G, rs137852313 T and rs1050829 C amino acid substitutions result in a stabilising effect.

### High variability of rs1050828 allele frequency in Africa

Previous studies have shown that the MAF of rs1050828 is relatively high in African populations [37, 38]. We further show that it is also extremely variable—even within the same geographical region. Table 2 shows the MAF in the AWI-Gen study (over 11,030 participants). A full per-group analysis is not possible in this rapid communication. However, we show the MAF in selected groups with at least 180 individuals.

**Table 2:** Minor allele frequency (MAF) of rs1050828 (T) in selected groups from the AWI-Gen study genotype data.

Group	All *N*	MAF	Males MAF	*N*	Females MAF Het		Hom
All genotyped samples	11,030	12.6	11.9	6033	12.8	21.3	2.2
South Africa							
Tsonga BaPedi-Tswana-Sotho Xhosa	2132 1849 180	16.0 5.5 0.8	14.1 5.1 0.9	1209 1233 68	16.7 5.6 0.7	26.9 10.5 1.5	3.2 0.4 0.0
Ghana and Burkina Faso							
Mampruga-Mossi-Gouronsi-Kassena-Nankana	3723	18.6	17.6	1888	19.1	30.7	3.7
Kenya							
Luhya-Luo-Kamba Kikuyu	978 655	11.0 6.8	8.6 6.3	454 434	12.3 6.9	20.7 12.4	2.0 0.7

Het: Proportion of females heterozygous (%), Hom: Proportion of females homozygous for the alternate allele (%). Note that 100 of the HAAD SA individuals are included in this genotyping study (*<* 2% of the samples).

The rs1050828 T variant frequency in 11,030 individuals was 12.6%, close to the values reported in gnomAD (11.6%) and KGP (13.5%). Overall, 11.9% of African males carried the variant on their X chromosome while 2.2% of females were homozygous for the T allele. A moderate *G6PD* deficiency (10–60% residual enzyme activity) (WHO Class III) is likely to be present in individuals with such genotypes [13]. Although rare, the deficiency can present in heterozygous females depending on X-inactivation effects [9]. There was a significant difference in the variant frequency among self-identified ethnic groups in South Africa and Kenya. The frequency among the Tsonga was 16.0% which is substantially different from 0.8% found in the Xhosa (*p* = 2.4 × 10^*−*3^) and 5.5% in the BaPedi-Tswana-Sotho (*p* = 2.2 × 10^*−*16^) ethnolinguistic groups. The rs1050828 T variant appears at markedly different frequency in different Kenyan groups – 6.8% in the Kikuyu and 11% in the combined Luhya-Luo-Kamba groups (*p* = 4.3 × 10^*−*3^).

We tested for deviation from Hardy–Weinberg equilibrium (HWE) in the females in each of the groups except for the Xhosa (in which there was only 1 person who had the variant allele) and no significant deviation could be shown (lowest *p*-value was 0.38). In women overall the expected proportion of women who are heterozygous under assumption of HWE is 0.224 while the observed proportion was 0.213. While this is highly significant (*p* = 2.7 *×* 10^*−*4^), this deviation is not unexpected given the very significant differences in MAF between groups.

## Discussion

Current clinical studies that have used CQ/HCQ for treatment of COVID-19 have not explicitly taken into account the potential risks posed by G6PD deficiency [39–41]. G6PD deficiency is known to be common in Africans. In the present study, we assessed *G6PD* gene variation in African populations, and noted the high prevalence of a common deleterious allele—rs1050828 T. Although common, there are large differences in frequency for this variant, even between populations that are geographic neighbours. The differences may be explained by selective pressures in regions where malaria is/was common [42], as G6PD deficiency may convey resistance to malaria [43]. Such differences have been previously reported to occur even within countries, as in Botswana, where a decreasing trend in frequency of this variant occurs from the north-west to south-east [44]. Another study in South Africans (*n* = 181) from the Mpumalanga province reported the allele frequency of A– to be 14% [45], which is similar to our findings from AWI-Gen genotype data, where a MAF of 16% was found in Tsonga-speaking individuals. However, other SA groups, such as the Xhosa in particular (MAF 0.8%) had much lower frequencies. This highlights the limitation of reporting allele frequency by country rather than ethnolinguistic groups. African populations undergoing COVID-19 CQ/HCQ treatment trials may not have the same relative frequency of this allele as others. Thus, we urge that models for *G6PD*-related effects based on a single proxy African population are not directly transferable to other Africans, even those close geographically.

We observed other potentially deleterious variants in the African populations we studied. For instance, rs34193178 G and chrX:g.153762577 A > G, which were not found across all populations, do not have well characterised effects, and are unlikely to be included in assays to type well known *G6PD* variants. If these have a functional impact on G6DPD, they may add complexities to studies assessing the presence of rs1050828 T and rs1050829 C alone. The chrX:g.153762577 A > G variant in particular has structural evidence for deleterious functional impact.

Although use of either CQ/HCQ is not new in African populations, the dosage and duration of CQ/HCQ treatment for COVID-19 may lead to higher prevalence of adverse effects related to G6PD deficiency. Acute haemolysis was first observed in a G6PD deficient male suspected of carrying the *G6PD* Mediterranean variant (rs5030868 A) who was treated with lopinavir and HCQ [19]. Shortly thereafter, acute haemolysis was observed in two males of African ancestry—one of whom was treated with HQC [20], and another who also developed methemoglobinemia following treatment with CQ [21]. The case described in Kuipers et al. carries the A- variant (confirmed by genetic testing). These observations of haemolytic effects highlight the relevance of assessing the impact of G6PD deficiency on CQ/HCQ that is repurposed for COVID-19 treatment, particularly in African populations. Non-haemolytic adverse effects have also been noted in other recent trials. A CQ trial in Brazilians noted severe adverse reactions related to QT elongation [39], and the high doses (600 mg twice daily) may pose greater risks for individuals with G6PD deficiency. The proportion of African admixture for the patients assessed in this study was not disclosed. A recent trial in U.S. veterans showed increased risk of mortality in patients treated with HQC [46]. In addition, it is currently unknown how G6PD deficiency may affect COVID-19 disease progression. G6PD deficient cells have been found to be more vulnerable to human *alphacoronavirus* 229E infection in vitro, which correlated with elevated oxidant production [47], although it is not yet known if this effect would also be seen with the novel SARS-CoV-2 virus. Monitoring of G6PD deficiency throughout COVID-19 trials and studies in Africans may therefore also reveal other factors which are not limited only to effects of drug response. Such studies could make use of a rapid enzymatic assay for G6PD deficiency in lieu of DNA-based assays. These are available, though it should be noted that their sensitivity is lower in females [48].

As a final note, the applicability and evidence-base for CQ/HCQ as COVID-19 treatments have recently been reviewed [1, 49]. These reviews conclude that there is currently insufficient evidence for the use of CQ/HCQ as effective COVID-19 treatments. To date (February 2021), the debate about the efficacy of HQC in the treatment of COVID-19 patients is continuing with many conflicting reports [50, 51]. As additional randomised controlled trials have been recommended [1], we urge that G6PD deficiency-related effects be considered in African participants in these studies.

## Conclusion

Given our findings of the large heterogeneity of the *G6PD* gene, variants associated with G6PD deficiency in Sub-Saharan Africa, and the possible presence of other uncharacterised deleterious variants, it is important to consider the potential impact of these variants before widespread use of CQ/HCQ as COVID-19 treatments for African populations. Distinct African ethnolinguistic groups can have vastly different frequencies of G6PD deficiency, thus clinical studies of CQ/HCQ for COVID-19 in Africans should be conducted on diverse African populations, and with monitoring for haemolysis and/or anaemia. Targeted sequencing of *G6PD* among study participants would provide important insights into the risks of adverse effects at therapeutic doses which might lead to dosage adjustment.

## Data Availability

Datasets that were used in this work are available in the European Genome-phenome Archive (EGA). Human Heredity and Health in Africa (H3Africa) submissions to EGA that were used include: EGAD00001006418, EGAD00001004220, EGAD00001004448, EGAD00001004505, EGAD00001004533, EGAD00001004557, EGAD00001004393. Cell Biology Research Lab data submission: EGAD00001007589. HAAD includes other datasets: Simons Foundation Genome Diversity Project (EGAS00001001959) and from African collaborators—Southern African Human Genome Project (EGAS00001002639).
